# IgA Nephropathy: An Overview of the Clinical Trials

**DOI:** 10.1016/j.xkme.2025.101078

**Published:** 2025-08-05

**Authors:** Zohreh Gholizadeh Ghozloujeh, Haresh Selvaskandan, Nasim Wiegley, Edgar Lerma, Jorge Gaytan, Alejandro Garcia-Rivera, Amir Abdipour, Sayna Norouzi

**Affiliations:** 1 Department of Medicine, Division of Nephrology, Loma Linda University, Loma Linda, CA; 2 John Walls Renal Unit, University Hospitals Leicester NHS Trust, Leicester, UK; 3 Department of Medicine, Division of Nephrology, University of California Davis School of Medicine, Division of Nephrology, Sacramento, CA; 4 Department of Medicine, Section of Nephrology, Advocate Christ Medical Center, University of Illinois at Chicago, Oak Lawn, IL; 5 Division of Medicine, Department of Internal Medicine and Nephrology, High Specialty Medical Unit No. 71, Mexican Social Security Institute (IMSS), Torreon, Mexico; 6 Department of Nephrology, Regional High Specialty Hospital, Institute for Social Security and Services for State Workers (ISSSTE), Torreon, Mexico; 7 Faculty of Medicine, Laguna Unit, Autonomous University of Coahuila, Torreon, Mexico; 8 Department of Nephrology, Hospital General Regional 46, Instituto Mexicano del Seguro Social, Guadalajara, Mexico

**Keywords:** Clinical trials, IgA nephropathy, immune regulation, novel therapeutics

## Abstract

IgA nephropathy (IgAN) is characterized by the deposition of poorly-*O*-galactosylated IgA1 (also referred to as galactose-deficient IgA1 or gd-IgA1) containing immune complexes in the glomerular mesangium. This triggers a variable degree of glomerular inflammation that leads to progressive kidney damage and often kidney failure. The acceptance of proteinuria reduction as a reasonably likely surrogate end point for treatment effects on progression to kidney failure has led to many clinical trials evaluating novel and repurposed therapies for IgAN. New treatments leverage different aspects of IgAN pathophysiology, including the modulation of mucosal immunity, mechanisms of B-cell activation, and complement activity. The approval of the first treatments evaluated specifically for IgAN (including delayed-release budesonide, sparsentan, atrasentan, and iptacopan) represent meaningful advancements in the management landscape, and promisingly, many more treatments seem poised to arrive. This review compiles a list of current active trials for IgAN and highlights the necessity for ongoing research to optimize therapeutic strategies to further improve outcomes for those living with IgAN.

## Background

**Figure 1:**
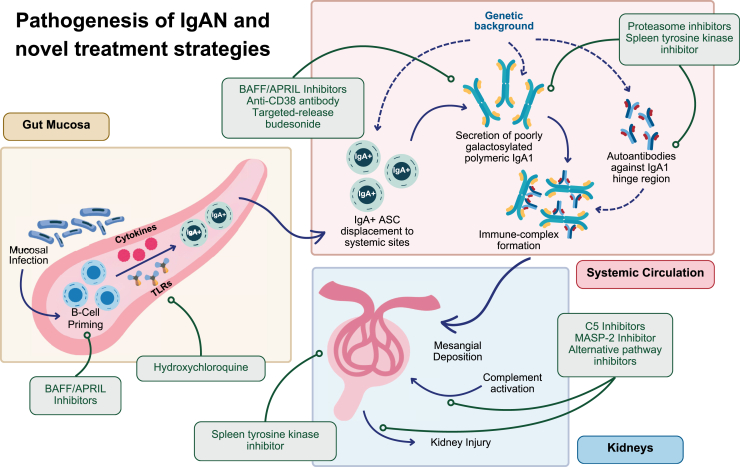
Pathogenesis of IgAN and novel treatment strategies. APRIL, A proliferation-inducing ligand; ASC, antibody-secreting cells; BAFF, B-cell activating factor; MASP-2, mannan-binding lectin-associated serine protease 2; TLR, toll-like receptor.

IgA nephropathy (IgAN) is the most commonly reported primary glomerulonephritis worldwide.^1^ It is most frequently diagnosed in young adults, many of whom are at risk of progressing to kidney failure within their lifetime, even at lower levels of proteinuria.^2^^,^^3^ The pathophysiological pathway of primary IgAN is multifactorial, with each element likely to play a variably important role in different patients, accounting for its particularly heterogenous clinicopathological presentation. The appearance of poorly-*O*-galactosylated IgA1 in the serum appears to be an important event in the pathophysiology of IgAN and may variably occur due to dysregulations in the mucosal microbiome, mucosal immune system, and B-lymphocyte activation, homing and subsequent antibody production (Fig 1).^4^^,^^5^ Gd-IgA1 forms immune complexes in the serum, which get deposited in the glomerular mesangium, triggering an inflammatory response mediated by cytokines, the complement system, and infiltrating inflammatory cells.^4^ These events alter glomerular hemodynamics, damage the glomerulus and surrounding tubules, and lead to proteinuria, which can also directly damage tubules.^2^^,^^4^^,^^6^ Each of these contributors appear to be mediated by both environmental and genetic influences, and each have the potential to be manipulated for therapeutic benefit.^4^

Clinical trials for IgAN have long been hindered by the slowly progressive nature of the condition. The acceptance of proteinuria reduction as a reasonably likely surrogate end point for treatment effects on progression to kidney failure for IgAN transformed the clinical trial landscape for IgAN by making trials more feasible.^7^ This has led to vast number of clinical trials evaluating novel and repurposed therapies for IgAN, leading to the first ever treatments to receive Food and Drug Administration (FDA) approval for the management of IgAN. These treatments include delayed-release budesonide (DR-budesonide) and sparsentan, both of which have now received full FDA approval, as well as atrasentan and iptacopan, which have received accelerated approval pending confirmatory evidence from ongoing phase 3 trials. Accelerated approval is typically granted based on surrogate endpoints such as proteinuria reduction, with full approval contingent on demonstrating long-term kidney benefit, often assessed using changes in estimated glomerular filtration rate (eGFR) slope over 2 years.^8^, ^9^, ^10^, ^11^, ^12^, ^13^, ^14^, ^15^ Many more therapies are currently being evaluated for IgAN, making emergent treatments for IgAN a rapidly advancing field.^16^, ^17^, ^18^, ^19^ This review offers a narrative overview of recent and active clinical trials investigating emerging treatment options for IgAN, highlighting key themes and insights within the current literature.

## Methods

We conducted a comprehensive search using the ClinicalTrials.gov advanced search function to identify IgAN related clinical trials from January 2014 to January 2025. (1) Filters applied included the following: condition = “IgA nephropathy,” study type = interventional, study phase = 2 or 3, recruitment status = completed, recruiting, or active not recruiting, and population age ≥18 years. (2) From the filtered results, we excluded studies that had not yet started recruiting, were terminated, enrolled pediatric-only populations, or involved drugs lacking information on pharmacokinetics, pharmacodynamics, or mechanism of action. We focused on trials evaluating novel therapeutic interventions with mechanistic relevance. From the final list of eligible studies, we extracted data on interventions, study design, primary endpoints, and trial status.

## Results

We identified a total of 44 interventional clinical trials for IgAN. Among these, 34 specifically focused on treatments for IgAN and met our criteria by reporting primary outcome measures relevant to modifying prognosis for those with IgAN (Table 1).

**Table 1:** Summary of Phase 2 and 3 Clinical Trials Investigating Emerging Therapies for IgA Nephropathy, Grouped by Drug Class and Mechanism

Drug Class	Drug and Trial (NCT ID)	Therapeutic Target	Trial Phase	Status	Primary Endpoint(s)	Key Results
Endothelin receptor antagonists	Sparsentan (NCT03762850/ PROTECT)	Dual endothelin A + angiotensin II receptor antagonist	Phase 3	Completed	% change in UPCR at wk 36	49.8% ↓ in UPCR vs irbesartan; sustained eGFR
	Sparsentan (NCT04663204/ SPARTAN)		Phase 2	Active	Change in UPCR at wk 36	Ongoing
	Sparsentan (NCT05856760/ SPARTACUS)		Phase 3	Recruiting	Proteinuria reduction + kidney preservation	Ongoing
	Atrasentan (NCT04573478/ALIGN)	Selective endothelin A receptor antagonist	Phase 3	Active	Change in 24-h UPCR at wk 36, eGFR over 136 wk	38.1% ↓ in UPCR; no heart failure/edema increase
	Atrasentan (NCT05834738/ ASSIST)		Phase 2	Active	UPCR changes at wk 12 and 24 (with SGLT2i)	Ongoing
	SC0062 (NCT05687890)	Selective endothelin receptor type A antagonist	Phase 2	Active, not recruiting	UACR, UPCR, eGFR changes; % of subjects with ≥30% to 50% proteinuria reduction	Ongoing dose-finding trial; safety and efficacy in IgAN
Systemic immunosuppressants	Prednisolone ± Cyclophosphamide (NCT05510323)	Immune suppression via corticosteroids ± alkylating agent	Phase 3	Active	40% ↓ in eGFR, kidney failure, or death from kidney disease over 6 y	Ongoing
	Corticosteroids ± RAAS/SGLT2i (NCT03188887/ TIGER Study)	Immune suppression with background RAAS or SGLT2i in patients with severe histologic lesions	Phase 3	Completed	Composite kidney failure endpoints at 6, 12, and 24 mo; repeat biopsy histology changes	Results pending; evaluates benefit of adding corticosteroids to supportive care
Locally acting corticosteroids	Nefecon (NCT03643965/ NEFIGARD)	Mucosal immune modulation via inhibition of Gd-IgA1 formation	Phase 3	Completed	UPCR, eGFR changes at 9 mo and 2 y	↓ UPCR; eGFR stabilization
	Nefecon (NCT04541043/NEFIGARD OLE)		Phase 3b	Active	UPCR, eGFR at 9 mo; safety at 12 mo	Ongoing
	Nefecon (NCT05534919)		Phase 3b	Active	UPCR and eGFR at 9 mo; safety follow-up to 12 mo	Ongoing
Immunomodulator	Hydroxychloroquine sulfate (NCT02942381)	Immune modulation via TLR7/TLR9 inhibition and cytokine suppression	Phase 2	Completed	Proteinuria reduction and kidney function stabilization over 6 mo	↓ Proteinuria; eGFR stable; well tolerated
B-cell modulators	Atacicept (NCT04716231)	Dual BAFF/APRIL inhibition	Phase 2b/3	Active	Change in UPCR from baseline at 36 wk	↓ UPCR; eGFR stabilized; good safety profile
	Atacicept extension (NCT06674577)		Phase 2	Recruiting	Long-term safety; changes in UPCR, eGFR, hematuria, Gd-IgA1	Extension of Vera trial; results pending
	Telitacicept (NCT05799287)		Phase 3	Active	Change in proteinuria from baseline to wk 24	Ongoing
	Telitacicept (NCT04291781)		Phase 3	Active	Long-term proteinuria reduction and kidney function preservation	Ongoing
	Povetacicept (NCT06564142)	Dual BAFF/APRIL inhibition (enhanced TACI-Fc fusion protein)	Phase 3	Active	Change in 24-h UPCR at wk 36; eGFR and fatigue at wk 104	↓ UPCR; eGFR stable; well tolerated (RUBY-3)
	BION-1301 (NCT05852938)	APRIL inhibition to reduce Gd-IgA1	Phase 3	Active	Change in UPCR from baseline to wk 40	Ongoing
	BION-1301 (NCT03945318)		Phase 1/2	Completed	Safety, tolerability, PK/PD, TEAEs	↓ Gd-IgA1, ↓ proteinuria; no serious AEs
	Sibeprenlimab (NCT04287985)	APRIL-neutralizing monoclonal antibody	Phase 2	Completed	Change in UPCR, 24-h urine protein, safety	↓ APRIL, ↓ Gd-IgA1; well tolerated
	Sibeprenlimab (NCT05248646/ Visionary)		Phase 2	Active	Change in UPCR over 9 mo; eGFR slope over 24 mo	Ongoing
	Sibeprenlimab (NCT06740526)		Phase 2b	Recruiting	Change in glomerular IgA deposition by IF (wk 52)	Histopathology driven; safety and tissue response focus
Spleen tyrosine kinase (Syk) inhibitors	Fostamatinib (NCT02112838)	Inhibition of Syk-mediated inflammation and antibody signaling	Phase 2	Completed	Change in proteinuria, glomerular sclerosis, and eGFR over 24 wk	↓ Proteinuria; stabilized kidney function
CD38 inhibitors	Felzartamab (NCT05065970/ IGNAZ)	Depletion of CD38+ plasma cells (IgA1 producers)	Phase 2	Active	Complete response rate, pharmacokinetics, serum IgA and Gd-IgA1 levels	↓ UPCR; stable eGFR; well tolerated
Complement inhibitors	Iptacopan (NCT03373461)	Factor B inhibition (alternative pathway)	Phase 2	Completed	UPCR reduction over 90 d	Dose-dependent ↓ proteinuria (esp. 200 mg twice a d)
	Iptacopan (NCT04557462)		Phase 2	Active	Sustained UPCR ↓, eGFR preservation	Ongoing
	Iptacopan (NCT04578834)		Phase 3	Active	UPCR at 9 mo, annualized eGFR slope over 24 mo	Ongoing
	HRS-5965 (NCT06137768)	Alternative and lectin complement pathways	Phase 2	Recruiting	UPCR change from baseline (wk 12 and 24); eGFR and serum creatinine change	Ongoing; developed for PNH, now in IgAN evaluation
	Avacopan (NCT02384317)	C5a receptor blockade (complement inhibition)	Phase 2	Completed	Proteinuria reduction, kidney function stability	↓ proteinuria; maintained eGFR; well tolerated
	Ravulizumab (NCT04564339)	Terminal complement (C5) inhibition	Phase 2	Completed	% change in proteinuria at wk 26 and 50; eGFR changes	↓ 30.1% in proteinuria; eGFR trend stable
	Ravulizumab (NCT06291376/ICAN)		Phase 3	Active	UPCR, eGFR, fatigue, composite kidney failure at 106 wk	Ongoing
	IONIS-FB-LRx (NCT04014335)	Antisense oligonucleotide against CFB mRNA	Phase 2	Completed	% ↓ in 24-h proteinuria, albuminuria, plasma CFB	↓ proteinuria; ↓ CFB; safe and well tolerated
	RO7434656 (NCT05797610/ IMAGINATION)	Antisense inhibitor of complement factor B	Phase 3	Active	UPCR at wk 37; eGFR slope; composite kidney failure	Ongoing
Melanocortin receptor agonists	ACTH Gel (Acthar) (NCT02282930)	Immunomodulation via melanocortin receptor activation	Phase 3	Completed	% of patients achieving complete or partial remission at 12 mo (based on proteinuria and GFR criteria)	↓ proteinuria (median 2.6 → 1.3 g/24 h); eGFR stable; 42% achieved partial remission. Mild AEs: depression, anxiety, injection reactions, 6 infections (none requiring hospitalization)

CFB, complement factor B; SGLT2i, sodium-glucose co-transporter 2 inhibitor; TACI, transmembrane activator and CAML interactor; PNH, paroxysmal nocturnal hemorrhage.

These selected studies primarily assessed the efficacy and safety of targeted treatments. Primary endpoints usually included proteinuria reduction, measured through the urine protein-to-creatinine ratio (UPCR) or albumin-to-creatinine ratio, and stabilization or improvement in eGFR slopes, over specific timeframes. Adverse events were frequently reported to provide safety profiles for each intervention. Secondary endpoints commonly evaluated additional markers of kidney health, including changes in eGFR, rate of eGFR decline, and composite metrics like time to kidney failure. Table 2 summarizes trials by targeted therapeutic mechanism, illustrating the diversity of approaches under investigation for IgAN management.

**Table 2:** Distribution of IgA Nephropathy Interventional Trials by Targeted Therapeutic Mechanism

Mechanism Targeted	Number of Trials	Description
Endothelin receptor antagonists	6	Trials targeting the RAS system and endothelin receptors to reduce Proteinuria and preserve kidney function, including agents like sparsentan, atrasentan, and SC0062.
Corticosteroids and immunosuppressants	2	Studies on corticosteroids, alone or with immunosuppressants, for high-risk IgAN cases to reduce proteinuria and manage severe disease progression.
Targeted release budesonide	3	Trials focusing on budesonide to lower Gd-IgA1 formation and reduce proteinuria, aiming to preserve long-term kidney function.
Immunomodulatory agents (Nonsteroidal)	1	Research on hydroxychloroquine to reduce proteinuria in patients unresponsive to conventional treatments, improving kidney function stability.
B-cell Modulators	10	Trials targeting B-cell activity with drugs like atacicept, telitacicept, povetacicept, BION-1301, and VIS649 to reduce pathogenic IgA1 and proteinuria.
Spleen tyrosine kinase (Syk) inhibitors	1	Fostamatinib is tested to inhibit Syk activity, aiming to reduce proteinuria and stabilize kidney function.
CD38 inhibitors	1	Felzartamab targets CD38-positive plasma cells, aiming to lower Gd-IgA1 and reduce proteinuria for kidney stability.
Complement inhibitors	9	Trials targeting the complement pathway using agents like LNP023, CCX168, ravulizumab, and others to reduce proteinuria and kidney inflammation.
Melanocortin receptor agonists	1	One trial investigating ACTH gel for its anti-inflammatory and immunomodulatory effects in IgAN. With modest reduction in proteinuria, its clinical role remains investigational.

## Discussion

### Endothelin Receptor Antagonists

Endothelin receptor antagonists (ERAs), particularly those targeting the endothelin A receptor (ETAR), have shown promise in reducing proteinuria and mitigating kidney deterioration in IgAN. These agents counteract ETAR-mediated vasoconstriction, inflammation, cell proliferation, and fibrosis, which contribute to progressive glomerular damage.^20^^,^^21^

#### Sparsentan

Sparsentan, a dual angiotensin and endothelin receptor antagonist, demonstrates greater proteinuria reduction in IgAN than in focal segmental glomerulosclerosis.^22^ Its mechanism of action involves blockade of the angiotensin II type 1 receptor and the ETAR, both of which are implicated in glomerular injury and proteinuria.^23^ In 2024, sparsentan received full FDA approval for adults with IgAN at risk of disease progression.^17^

#### Atrasentan

Atrasentan is a selective ETAR antagonist that has received accelerated FDA approval based on interim results from the ALIGN trial.^12^^,^^24^^,^^25^ Preclinical studies indicate that atrasentan restores the glomerular endothelial glycocalyx and attenuates mesangial injury and profibrotic signaling.^26^^,^^27^ In a cohort of 17 IgAN patients, adding atrasentan to standard of care led to a marked reduction in proteinuria after 12 weeks.^28^ The therapy was well tolerated, underscoring its potential as a promising treatment option in IgAN.^28^

#### SC0062

SC0062 is a next-generation selective ETAR antagonist that preserves endothelin B receptor signaling, potentially minimizing fluid retention while maximizing antiproteinuric and antifibrotic effects.^29^, ^30^, ^31^ In phase 1 trials, SC0062 demonstrated dose-proportional pharmacokinetics, favorable tolerability, and biological activity through suppression of endothelin-1-induced pressor responses.^30^

#### Efficacy, Safety, and Treatment Considerations

Sparsentan and atrasentan have been studied in both monotherapy and combination settings (eg, RAS inhibitors and/or SGLT2 inhibitors), whereas SC0062 remains in early development (Table 1).

Among the ERAs, in the PROTECT trial, sparsentan demonstrated a 49.8% reduction in proteinuria at week 36 versus 15.1% with irbesartan (*P* < 0.0001), with a more favorable chronic eGFR slope and similar safety profile aside from slightly higher peripheral edema.^32^ An interim update also confirmed the sustained antiproteinuric effect and statistically significant benefit in chronic eGFR slope over 110 weeks.^33^ In SPARTAN, an open-label trial, newly diagnosed, treatment-naïve patients experienced a 62.8% reduction in proteinuria with sparsentan and generally mild to moderate adverse events, with dizziness and one withdrawal because of hypotension being the most notable.^34^

Atrasentan, evaluated as an adjunct to angiotensin-converting enzyme inhibitors or angiotensin II receptor blockers in the ALIGN trial, showed a 38.1% proteinuria reduction at week 36 compared to 3.1% with placebo (*P* < 0.001), with no excess risk of heart failure or severe edema.^35^ The trial included an exploratory sodium-glucose co-transporter 2 inhibitor stratum to assess dual-pathway synergy, reflecting growing interest in dual-pathway blockade in IgAN.^36^ SC0062, still in phase 2 development, demonstrated over 40% reduction in proteinuria at the 10 mg dose, with over half of treated patients achieving ≥30% reduction. It was generally well tolerated, with low incidence of edema and no serious fluid-related events.^29^

Among ERAs, sparsentan and atrasentan have demonstrated robust antiproteinuric effects and potential for kidney preservation. Sparsentan’s renin–angiotensin system (RAS)-replacement model offers a streamlined mechanism of action but may carry a slightly higher risk of edema and hyperkalemia. Unlike sparsentan, atrasentan does not require a risk evaluation and mitigation strategy program or monthly liver function testing, whereas sparsentan remains subject to risk evaluation and mitigation strategy requirements, including monthly liver enzyme and pregnancy monitoring.^23^ Atrasentan’s adjunctive use with angiotensin-converting enzyme inhibitor or angiotensin II receptor blocker, and its incorporation of an sodium-glucose co-transporter 2 inhibitor arm, allows a more modular approach to therapy. SC0062, although promising, requires further validation in larger trials. Overall, ERAs represent an evolving class with strong mechanistic rationale, encouraging efficacy data, and emerging strategies for combination use. Although ERAs benefit patients with IgAN, they are unlikely to directly target disease-specific mechanisms such as Gd-IgA1 production or mesangial deposition. The ongoing SPARTAN trial may clarify their role in early-stage immunopathology and broader therapeutic relevance.

Further investigation will be essential to clarify long-term outcomes, cardiovascular safety, and optimal patient selection for this class.

### Adrenocorticotropic Hormone

Adrenocorticotropic hormone **(**ACTH,) a melanocortin peptide hormone, exerts anti-inflammatory and podocyte-protective effects via melanocortin receptor activation. Its role in IgAN is under investigation, with limited data from early-phase studies and small prospective cohorts.^37^, ^38^, ^39^

#### Efficacy, Safety, and Treatment Considerations

In an open-label trial (NCT02282930), ACTH led to a significant reduction in proteinuria from a median of 2.6 to 1.3 g/24 h over 12 months (*P* = 0.007), with stable eGFR and 42% achieving partial remission. Reported adverse events were mild but included depression, anxiety, injection reactions, and 6 infections of 19 (none requiring hospitalization).^40^

ACTH is not recommended in Kidney Disease: Improving Global Outcomes 2024 guideline due to limited supporting evidence, uncertain benefit, and high cost.^14^ Although small studies suggest benefit in proteinuria, its role remains investigational and it has been employed in refractory cases, though supporting evidence is limited.^37^^,^^39^

### Corticosteroids

Corticosteroids have long been used to reduce proteinuria and slow kidney function decline in patients with IgAN, particularly those at high risk of progression with persistent proteinuria (>1 g/day) despite optimized RAS blockade.^14^^,^^41^^,^^42^ Prednisolone remains the most widely studied agent. Its mechanism of benefit relates to anti-inflammatory and immunosuppressive effects, but long-term toxicity concerns especially in patients with advanced chronic kidney disease have limited broad uptake.^42^

Prednisolone in combination with RAS inhibitors has also been shown to effectively reduce proteinuria and preserve kidney function in IgAN patients.^14^^,^^43^ Combination therapy with cytotoxic agents (eg, cyclophosphamide) is typically reserved for rapidly progressive glomerulonephritis IgAN patients (defined as >50% decline in eGFR within 3 months).^14^^,^^44^^,^^45^ Increasingly, lower-dose and shorter-duration steroid regimens are adopted to mitigate adverse events while preserving therapeutic effect; however, this approach remains under investigation.^43^

#### Efficacy, Safety, and Treatment Considerations

Recent trials have aimed to refine corticosteroid use across IgAN severities. One ongoing study (NCT05510323) is comparing low-dose oral corticosteroids with or without cyclophosphamide in patients with stage 3-4 chronic kidney disease, using hard endpoints such as 40% eGFR decline, kidney failure, or death over 6 years. Another completed trial evaluated early corticosteroid therapy based on histologic severity versus supportive care alone. The primary endpoint was a composite of proteinuria thresholds, GFR decline, or kidney failure at 24 months.

Corticosteroids remain a therapeutic option in IgAN, particularly for high-risk patients with persistent proteinuria.^14^ Despite evidence of efficacy, risks include infection, metabolic disturbances, and cardiovascular complications, especially with high-dose or prolonged use.^42^^,^^46^ Consequently, recent trials have shifted focus toward refining corticosteroid use through lower-dose protocols, shortened treatment durations, and improved patient selection strategies. These studies aim to define safer, more targeted approaches.

As a result, both guidelines and recent expert reviews emphasize individualized decision making, with supportive care forming the foundation of management and corticosteroids reserved for selected high-risk patients at low risk of adverse events based on risk-benefit assessment.^43^^,^^47^

### Delayed-release budesonide

DR-budesonide is through to reduce Gd-IgA1 production by modulating mucosal immune responses in the distal ileum, leading to reduced proteinuria and preservation of kidney function.^48^^,^^49^ As a result, it modulates kidney inflammation and fibrosis pathways and downregulates specific serum microRNAs.^50^^,^^51^

#### Efficacy, Safety, and Treatment Considerations

DR-budesonide has been evaluated in several trials. In the pivotal NEFIGARD study, it reduced UPCR by 27% at 9 months versus placebo (*P* = 0.0001) and slowed eGFR decline over 2 years (−2.47 vs −3.74 mL/min/1.73 m^2^/year; *P* = 0.029), suggesting long-term kidney protection.^48^^,^^52^ Adverse events were mostly mild to moderate (acne, moon face, hypertension), with low rates of serious events. In the NEFIGARD OLE, retreated patients sustained antiproteinuric effects, whereas delayed treatment patients showed a 36.4% median UPCR reduction and improved eGFR slope, reinforcing disease-modifying potential.^53^

DR-budesonide represents a novel approach by selectively targeting gut mucosal immune activation in IgAN, differentiating it from systemic immunosuppressants. Its efficacy in reducing proteinuria and preserving eGFR, with fewer systemic steroid-related toxicities, has been consistently observed in pivotal and extension trials.^52^^,^^53^ Kidney Disease: Improving Global Outcomes 2024 draft guidance positions DR-budesonide as an add-on therapy for adults with persistent proteinuria despite optimized RAS blockade and eGFR ≥35 mL/min/1.73 m^2^, favoring its use in high-risk, nonadvanced chronic kidney disease populations(14). However, questions remain regarding long-term efficacy beyond retreatment, optimal duration, its use in patients with low eGFR (<30 mL/min/1.73 m^2^), and its role in lower-risk or comorbid populations.^54^ Ongoing trials will help clarify its positioning, particularly its use in combination with sodium-glucose co-transporter 2 inhibitors and other emerging therapies, though data in lower eGFR populations remain limited.

### Hydroxychloroquine Sulfate

Hydroxychloroquine sulfate has shown effectiveness in reducing proteinuria in IgAN patients unresponsive to conventional therapy, primarily in Chinese cohorts.^55^^,^^56^ It modulates toll-like receptor signaling, inhibits proinflammatory cytokines, and may correct intestinal dysbiosis, reducing mucosal immune activation in IgAN.^55^^,^^57^^,^^58^ Hydroxychloroquine sulfate also effectively reduces proteinuria and microhematuria in select IgAN patients.^59^

#### Efficacy, Safety, and Treatment Considerations

Hydroxychloroquine sulfate has been evaluated in a single randomized controlled trial. Patients with persistent proteinuria (0.75-3.5 g/day) despite ≥3 months of optimized angiotensin-converting enzyme inhibitor or angiotensin II receptor blocker received hydroxychloroquine 200-400 mg/day, adjusted for eGFR. The study demonstrated reduced proteinuria and preserved kidney function.^60^ Adverse events were mild, mostly gastrointestinal, and no serious infections or retinopathy were reported.^60^

Hydroxychloroquine sulfate offers a well-tolerated, nonsteroidal option for patients with mild to moderate proteinuria who remain at risk despite supportive care.^59^^,^^61^ Compared with corticosteroids, it may provide similar proteinuria reduction with fewer adverse events.^56^^,^^60^ However, its generalizability outside of Chinese populations is uncertain, and it has not yet been evaluated in combination with newer therapies or in advanced chronic kidney disease. Broader global validation and longer-term outcome data are needed before wider adoption.

### B-Cell Modulators

B-cell modulators targeting B-cell activating factor (BAFF) and a proliferation-inducing ligand (APRIL) represent an expanding class of targeted therapies in IgAN.^62^^,^^63^ These agents modulate key steps in the generation and survival of B cells and plasma cells, reduce production of Gd-IgA1, and mitigate downstream immune complex formation.^64^, ^65^, ^66^ This class includes recombinant fusion proteins that simultaneously block BAFF and APRIL, as well as monoclonal antibodies that selectively neutralize APRIL.^62^^,^^63^ By intervening at the upstream immunologic level of the multi-hit IgAN pathogenesis model, these agents hold promise for disease modification in proteinuric patients at risk of progression.

#### Atacicept

Atacicept, a fusion protein targeting BAFF and APRIL, has shown promise in managing IgAN. Barratt et al reported a dose-dependent reduction of pathogenic factor Gd-IgA1 and improved proteinuria and kidney function.^65^

#### Telitacicept

Telitacicept also targets BAFF and APRIL and has shown efficacy in real-world and clinical studies from China.^67^^,^^68^ Non-randomized studies confirmed reductions in proteinuria and stable kidney function.^69^ The combination of telitacicept with low-dose mycophenolate mofetil has been proposed as a potentially effective treatment for IgAN.^70^ These studies collectively suggest that telitacicept may be an effective and safe treatment for IgAN.

#### Povetacicept

Povetacicept, a dual BAFF/APRIL antagonist, is an Fc fusion protein of a variant transmembrane activator and CAML interactor domain engineered for potent inhibition of B-cell activating factors.^71^ Povetacicept is currently being investigated for its therapeutic potential in IgAN and other autoantibody-associated glomerulonephritides.^71^ It has shown meaningful reductions in UPCR and serum IgA levels, with preserved kidney function and no severe infections or hypogammaglobulinemia reported in early clinical evaluation.^66^^,^^72^ This promising profile supports further investigation of povetacicept to confirm these benefits in a broader patient population.^66^^,^^71^

#### BION-1301

BION-1301 is a humanized IgG4 monoclonal antibody that neutralizes APRIL, thereby reducing the production of pathogenic Gd-IgA1, a key driver of IgAN pathogenesis.^63^ Interim phase 1/2 results show reduced Gd-IgA1 and proteinuria with no serious adverse events.^73^^,^^74^ The safety and tolerability of BION-1301 have been confirmed in nonclinical and phase 1 studies.^75^^,^^76^ The ongoing phase 3 trial will further evaluate its effect on proteinuria, eGFR, and clinical endpoints in IgAN patients.^63^

#### Sibeprenlimab (VIS649)

Sibeprenlimab is a fully human IgG2 monoclonal antibody that also targets APRIL, with a high specificity that enables selective suppression of IgA-producing B cells.^77^ Early clinical data demonstrated dose-dependent reductions in circulating APRIL, total IgA, Gd-IgA1, and IgM, while preserving vaccine-specific IgG responses (eg, severe acute respiratory syndrome coronavirus 2), highlighting a selective immunomodulatory profile.^77^, ^78^, ^79^ Despite the introduction of disease-specific treatments for IgAN, other potential therapeutic agents, such as VIS649, are supported by significant advances in understanding the disease's pathogenesis.^80^^,^^81^

#### Efficacy, Safety, and Treatment Considerations

B-cell modulators have been evaluated in several trials. In ORIGIN 3, atacicept significantly reduced proteinuria and stabilized eGFR over 36 weeks, with continued benefit at week 52 and no increase in serious infections or hypogammaglobulinemia.^82^ Elitacicept, evaluated in NCT04291781, also lowered proteinuria and maintained kidney function over 52 weeks, with remission rates up to 67% in Chinese cohorts and a favorable tolerability profile.^68^

APRIL inhibitors have shown encouraging early results. In a phase 1/2 trial, BION-1301 achieved a 57% reduction in UPCR over 76 weeks, with sustained eGFR and only mild, transient infections. Reductions were most notable for IgA and IgM.^83^ Sibeprenlimab (NCT04287985) similarly reduced proteinuria and stabilized kidney function, without major safety concerns, including infections.^77^

B-cell modulators differ in construct and trial maturity but share a goal of disrupting upstream immune drivers in IgAN. Dual BAFF/APRIL fusion proteins like atacicept and telitacicept show favorable efficacy and safety; however, telitacicept data derive mainly from Chinese cohorts, which may limit generalizability.^68^^,^^69^^,^^82^ Povetacicept may offer enhanced potency with minimal immunoglobulin depletion or infectious risk.^66^^,^^72^

APRIL-specific monoclonal antibodies appear to provide comparable proteinuria reduction with narrower immune targeting. Their selectivity may offer long-term safety advantages, particularly for patients at risk of broader immunosuppression.^73^^,^^77^^,^^78^

Dual blockade may offer broader immunologic control, whereas APRIL-only agents may represent safer options in select populations. Long-term kidney outcomes, global validation, and head-to-head trials will be key to future positioning.

### Spleen Tyrosine Kinase Inhibitors

Spleen tyrosine kinase (SYK) plays a key role in the pathogenesis of IgAN by mediating downstream signaling of immune complexes in mesangial cells, contributing to inflammatory cytokine production, mesangial proliferation, and glomerular injury.^84^, ^85^, ^86^ Studies have demonstrated that SYK inhibition may reduce proteinuria and stabilize kidney function in preclinical and clinical models of IgAN.^84^, ^85^, ^86^

#### Fostamatinib

Fostamatinib is an oral SYK inhibitor initially developed for autoimmune conditions. It has demonstrated anti-inflammatory effects by reducing autoantibody production, downregulating inflammatory cytokines, and reversing experimental glomerulonephritis, including IgAN.^84^^,^^86^^,^^87^

#### Efficacy, Safety, and Treatment Considerations

In the phase 2 fostamatinib trial, the overall cohort did not achieve a statistically significant reduction in proteinuria. However, at 24 weeks, a prespecified subgroup with UPCR >1000 mg/g demonstrated a dose-dependent reduction of 27% and 36% in the 100 mg and 150 mg groups, respectively, versus 14% with placebo. eGFR remained stable across all groups. Reported adverse events included diarrhea, elevated liver enzymes, and hypertension, with no serious infections observed.^84^

Fostamatinib offers a nonimmunosuppressive approach to IgAN through targeted inhibition of mesangial inflammation. Subgroup findings support potential benefit in high-risk proteinuric patients, although statistical significance was not achieved.^84^ Limitations include small sample size, modest duration, and baseline imbalances. Larger, longer studies are needed to validate efficacy and define its role in the IgAN therapeutic landscape.

### CD38 Inhibitors

CD38 inhibitors represent an emerging class of targeted therapies in IgAN. CD38 is highly expressed on long-lived plasma cells, which may contribute to the overproduction of pathogenic Gd-IgA1 and immune complexes. By depleting CD38^+^ plasma cells, these agents aim to reduce IgA-driven immune activity and mitigate kidney injury.^62^^,^^88^

#### Felzartamab

Felzartamab is a fully human anti-CD38 monoclonal antibody designed to deplete CD38+ plasma cells involved in the production of Gd-IgA1, while sparing other immune compartments. Early-phase studies have demonstrated durable reductions in circulating IgA and Gd-IgA1, with rapid IgG recovery and a favorable safety profile.^88^^,^^89^

#### Efficacy, Safety, and Treatment Considerations

In the IGNAZ trial, felzartamab led to a 35% reduction in UPCR at month 6 in the highest-dose group, compared to a 9% increase with placebo. The effect was sustained through month 15 despite treatment ending at month 5. eGFR remained stable in treated patients but declined in placebo. Reductions in serum IgA and Gd-IgA1 persisted ≥10 months post-treatment. Most adverse events were mild to moderate, including infusion reactions, fatigue, and transient cytopenia. No serious infections or hypogammaglobulinemia were reported.^89^

Felzartamab offers a novel mechanism compared to traditional immunosuppressants or B-cell modulators.^62^ The durable antiproteinuric effect observed beyond the treatment window and the selective immunoglobulin class suppression, with preserved IgG and no hypogammaglobulinemia support its potential as a disease-modifying agent.^89^ Its favorable safety profile, with no serious infections or long-term immune suppression signals, makes it a promising option for patients with refractory disease or those intolerant to broader immunosuppression.^89^ However, further studies are needed to validate efficacy in more diverse populations and assess long-term kidney outcomes.

### Complement Inhibitors

The complement system plays a central role in IgAN pathogenesis, particularly the alternative pathway (AP). Multiple agents now target distinct complement components, aiming to reduce immune complex-mediated glomerular damage.^62^^,^^90^

#### Iptacopan

Studies show that iptacopan, an oral complement AP factor B inhibitor, reduces proteinuria and slows kidney disease progression in IgAN patients.^90^^,^^91^ Iptacopan (LNP023) has been shown to improve lupus nephritis in animal models by inhibiting the activation of the alternative complement pathway.^92^ Other studies suggest that LNP023, particularly with RAS inhibitors, could be valuable in IgAN treatment.^93^^,^^94^

#### Avacopan

Avacopan (CCX168), a C5a receptor antagonist, has been shown to ameliorate anti-myeloperoxidase -induced necrotizing crescentic glomerulonephritis in murine models, supporting its mechanistic role in crescentic glomerulonephritis.^95^ Clinical trials in patients with anti-neutrophil cytoplasmic antibody-associated vasculitis have since demonstrated improved renal outcomes and reduced steroid burden with avacopan, the oral formulation of CCX168.^96^ Complement activation, particularly the role of C5a, has been implicated in the pathogenesis of IgAN, making CCX168 a potential target for therapy.^80^ Its pharmacologic and pharmacokinetic properties have been characterized, demonstrating safety and efficacy in early trials.^97^

#### Ravulizumab

Ravulizumab is a long-acting monoclonal antibody that inhibits terminal complement activation at C5.^98^ This drug provides immediate, sustained inhibition of the terminal complement pathway, critical in IgAN pathophysiology, aiming to reduce proteinuria and mitigate kidney function decline.^99^^,^^100^ Ongoing phase 2 and 3 studies substantiate the safety and potential benefits of ravulizumab, offering hope for a disease-modifying option in this challenging condition.^100^^,^^101^

#### IONIS-FB-LRx

IONIS-FB-LRx, an antisense oligonucleotide targeting complement factor B mRNA, has shown promise in reducing complement levels and proteinuria in IgAN patients.^102^ This RNA- interference therapy offers a targeted approach for upstream complement blockade and is currently being further evaluated.^102^^,^^103^

#### Sefaxersen

Sefaxersen (RO7434656), another antisense oligonucleotide inhibitor of complement factor B, has shown promising results in managing IgAN. A phase 2 trial demonstrated a reduction in UPCR and stabilization of the eGFR.^104^

#### HRS-5965

HRS-5965 is an oral small molecule that selectively inhibits MASP-2 and MASP-3, key activators of the alternative and lectin complement pathways, offering a novel upstream approach distinct from AP inhibitors.^62^ By modulating early complement cascade activation, HRS-5965 aims to reduce immune complex-mediated damage while preserving terminal complement function.^105^

#### Efficacy, Safety, and Treatment Considerations

Among agents inhibiting the AP, iptacopan demonstrated promising results in NCT03373461, significantly reducing proteinuria and improving eGFR slope over 9 months when combined with optimized supportive care, with a favorable safety profile and no serious adverse events attributed to the drug.^94^ Avacopan was evaluated in the phase 2 trial (NCT02384317). Although primarily studied in anti-neutrophil cytoplasmic antibody-associated vasculitis, early-phase studies showed potential kidney protection by mitigating complement-mediated inflammation. In IgAN, avacopan’s role remains exploratory, although its safety and tolerability have been established.^106^ In the SANCTUARY trial, ravulizumab provided sustained terminal complement blockade, with proteinuria reduction and stable kidney function over 52 weeks. Adverse events were infrequent, with no treatment-related serious events.^98^ RNA-based therapies targeting complement factor B include IONIS-FB-LRx, assessed in NCT04014335. This antisense oligonucleotide reduced plasma factor B levels and proteinuria with a favorable safety profile in IgAN patients over 29 weeks, supporting its role as a potential upstream regulator of AP activity.^102^ Similarly, sefaxersen (RO7434656), targeting the same mRNA, led to a mean 45% UPCR reduction and stable eGFR over 29 weeks in NCT05797610, with no drug-related serious adverse events reported.^104^

Complement inhibition offers a mechanistically precise strategy to counteract the glomerular injury central to IgAN. Iptacopan has emerged as the most clinically advanced oral option, showing rapid antiproteinuric effects and stable eGFR in short-term trials, with a favorable safety profile that supports long-term use.^94^ All patients in the phase 2 trial were vaccinated against encapsulated bacteria in accordance with the study’s protocol, and iptacopan is now subject to a risk evaluation and mitigation strategy program requiring vaccination against *Neisseria meningitidis*, *Streptococcus pneumoniae*, and *Haemophilus influenzae* type b before treatment initiation. Ravulizumab targets the terminal pathway and demonstrated sustained efficacy through week 50 in the SANCTUARY trial, without immunosuppression-related toxicity, positioning it as a compelling disease-modifying candidate.^98^ Antisense oligonucleotides such as IONIS-FB-LRx and Sefaxersen inhibit factor B synthesis upstream, offering durable reductions in complement activity and proteinuria with low immunologic burden.^102^^,^^104^ Compared to traditional immunosuppressive approaches, complement-targeted therapies offer a more selective, pathophysiology-driven mechanism with a potentially improved safety profile, especially when upstream components such as factor B are inhibited. AP blockade preserves terminal complement activity, minimizing infection risk while effectively dampening glomerular inflammation in IgAN.^62^^,^^107^ As these agents advance through pivotal trials, their integration into IgAN care will depend on comparative efficacy, kidney durability, safety, and pathway redundancy, particularly in combination with RAS or SGLT2 blockade.

## Conclusion

Recent clinical trials have provided encouraging insights into new treatment avenues for IgAN. These studies have highlighted the pivotal roles of mucosal immunity, B-cell activation, and mesangial cell activation in the disease’s progression. The approval of DR-budesonide, sparsentan, and the recent accelerated approval of iptacopan, along with the development of therapies targeting the mucosal immune system, B cells, the complement system, and the endothelin system, marks a significant advancement in the therapeutic landscape of IgAN. However, despite the recent advancements in therapeutic options and clinical trial design for IgAN, current therapies and studies have not yet adequately focused on specific patient subgroups. Critical gaps remain in understanding how treatment responses may differ based on factors such as sex, disease severity, comorbid conditions, and underlying histopathologic features. For instance, patients with rapidly progressive forms of IgAN, those with high chronicity scores on biopsy, or those belonging to underrepresented populations in trials may have distinct therapeutic needs that remain unaddressed. Future research should prioritize stratifying patients according to these characteristics to ensure that emerging therapies are tailored to the diverse clinical and pathological spectrum of IgAN, although we acknowledge that achieving this will require substantially larger studies and presents significant logistical challenges. This approach will be essential for advancing personalized medicine and improving outcomes across all patient populations.
