# Corrigendum to IgA Nephropathy: An Overview of the Clinical Trials (*Kidney Med.* 2025;7:101078)

**DOI:** 10.1016/j.xkme.2025.101132

**Published:** 2025-10-01

**Authors:** Zohreh Gholizadeh Ghozloujeh, Haresh Selvaskandan, Nasim Wiegley, Edgar Lerma, Jorge Gaytan, Alejandro Garcia-Rivera, Amir Abdipour, Sayna Norouzi

**Affiliations:** 1 Department of Medicine, Division of Nephrology, Loma Linda University, Loma Linda, CA; 2 John Walls Renal Unit, University Hospitals Leicester NHS Trust, Leicester, UK; 3 Department of Medicine, Division of Nephrology, University of California Davis School of Medicine, Division of Nephrology, Sacramento, CA; 4 Department of Medicine, Section of Nephrology, Advocate Christ Medical Center, University of Illinois at Chicago, Oak Lawn, IL; 5 Division of Medicine, Department of Internal Medicine and Nephrology, High Specialty Medical Unit No. 71, Mexican Social Security Institute (IMSS), Torreon, Mexico; 6 Department of Nephrology, Regional High Specialty Hospital, Institute for Social Security and Services for State Workers (ISSSTE), Torreon, Mexico; 7 Faculty of Medicine, Laguna Unit, Autonomous University of Coahuila, Torreon, Mexico; 8 Department of Nephrology, Hospital General Regional 46, Instituto Mexicano del Seguro Social, Guadalajara, Mexico

The authors regret that several changes to the text were not incorporated before publication. The necessary edits are listed below.

On page 9, the section “IONIS-FB-LRx” should instead have been titled “IONIS-FB-LRx (Sefaxersen).”

On page 9, in the section “IONIS-FB-LRx (Sefaxersen),” a citation to reference 104 should have been inserted alongside the citations to references 102 and 103 at the end of the sentence beginning “This RNA- interference therapy offers…”

On page 9, the section “Sefaxersen” should have been deleted entirely.

On page 10, a citation to reference 104 should have been inserted at the end of the sentence beginning “This antisense oligonucleotide reduced plasma factor B levels…”

On page 10, the sentence “Similarly, sefaxersen (RO7434656), targeting the same mRNA, led to a mean 45% UPCR reduction and stable eGFR over 29 weeks in NCT05797610, with no drug-related serious adverse events reported” should have been deleted.

On page 10, in the sentence “Antisense oligonucleotides such as IONIS-FB-LRx and Sefaxersen inhibit factor B synthesis upstream, offering durable reductions in complement activity and proteinuria with low immunologic burden,” the words “and Sefaxersen” should have been deleted.

The authors apologize for any inconvenience caused.

Zohreh Gholizadeh Ghozloujeh, MD, Haresh Selvaskandan, MD, Nasim Wiegley, MD, Edgar Lerma, MD, Jorge Gaytan, MD, Alejandro Garcia-Rivera, MD, Amir Abdipour, MD, and Sayna Norouzi MD

